# Trends and hot spots in research on the prognostic value of gastric cancer biomarkers in the context of the Lauren classification: a bibliometric analysis

**DOI:** 10.3389/fmed.2025.1612256

**Published:** 2025-07-22

**Authors:** Nurbek Kozhakhmetuly Azbergenov, Talshyn Amirkhanqyzy Nurulla, Anar Balkashevna Tulyayeva, Saule Zhumabaevna Akhmetova, Nurgul Meirimovna Kereeva, Saltanat Nurslyamovna Akanova, Dinara Almatovna Zholmukhamedova

**Affiliations:** ^1^Department of Pathological Anatomy and Forensic Medicine, West Kazakhstan Marat Ospanov Medical University, Aktobe, Kazakhstan; ^2^Department of Oncology, West Kazakhstan Marat Ospanov Medical University, Aktobe, Kazakhstan; ^3^Family Medicine Clinic, West Kazakhstan Marat Ospanov Medical University, Aktobe, Kazakhstan

**Keywords:** gastric cancer, gastric carcinoma, biomarker, biomarkers, Lauren classification, Lauren’s classification, prognosis

## Abstract

**Introduction:**

Modern research is aimed at finding reliable prognostic biomarkers. The Lauren classification remains an important tool for predicting outcomes in gastric cancer, especially when combined with certain biomarkers and tumor molecular characteristics. This study aims at quantifying the accumulated knowledge about the prognostic value of stomach cancer biomarkers in the context of the morphological Lauren’s classification, as well as providing recommendations for future studies.

**Methods:**

A literature search was conducted in the Scopus database in December 2024. The selection included 162 publications for the period 1995–2024. The analysis was performed using the statistical software RStudio, widely used in scientific research. The specialized Biblioshiny package was utilized for data analysis and visualization.

**Results:**

The average annual growth rate was 3.86%, and the average number of citations per article - 30.4, which underlines the high importance of the topic of the study. The largest number of publications originated from China (292 articles). Leading research institutions include Sungkyunkwan University School of Medicine (13 documents), Fudan University (12 documents), and Yonsei University College of Medicine (11 documents). The most prolific author is Zhang J., who conducted 13 studies in this field. The journal Pathology - Research and Practice holds the leading position with 8 publications. The most frequently occurring keyword is “gastric cancer” (94 mentions). Thematic trends include research on HER2 and microsatellite instability.

**Conclusion:**

The bibliometric analysis revealed the active development of research on the role of biomarkers in predicting gastric cancer prognosis based on Lauren’s classification. China is the leading country in this field, Sungkyunkwan University School of Medicine is the most active research institution, Zhang J. is the most influential researcher, Pathology - Research and Practice is the most productive journal. Current research focuses on HER2 and microsatellite instability.

## 1 Introduction

Gastric cancer (GC) is one of the most common and lethal oncological diseases of the gastrointestinal tract, accounting for 6.8% of the 20 million new cancer cases in 2022 (1). It is diagnosed twice as often in men (2) and is more prevalent in low- and middle-income countries, particularly in East and Central Asia (2–6). The main risk factors for developing GC include age, male sex, *Helicobacter pylori* infection, non-white race, smoking, socioeconomic status and dietary habits (4, 7). In advanced stage of disease, the median overall survival does not exceed 12 months (8). Overall five-year survival is approaching 28%, while the median five-year survival rate for late-stage disease is only 20% (9). The effectiveness of treatment and prognosis of GC largely depend on early diagnosis and disease progression assessment (10), which are complicated by the insufficient understanding of GC molecular mechanisms and the lack of reliable prognostic biomarkers (11, 12). Most cases are detected at late stages, reducing the effectiveness of therapy (13). Despite advances in treatment methods (robotic surgery, laparoscopy, multimodal therapy), no significant improvement in prognosis has been observed (14, 15). One of the reasons remains the lack of reliable biomarker panels for early detection, prognosis and stratification of patients (12). In this regard, the search for new reliable prognostic biological markers remains a pressing clinical challenge.

Modern studies aimed at assessing the expression of biomarkers and their correlation with clinical and morphological characteristics (11, 16, 17). In recent decades, the Lauren’s histological classification, proposed by him in 1965, has been most frequently used in studies (18). It is based on histopathological features and divides gastric cancer into three types: intestinal; diffuse; mixed (indeterminate) (18–20). A modified version of Lauren’s classification incorporates molecular and genetic tumor characteristics (21). It has been established that the intestinal type is more common in men and elderly patients, has a long course. The diffuse type is typical for young people and women, is associated with genetic factors and has a worse prognosis, is more often observed in Western countries, has a relatively low chemosensitivity (22, 23). The mixed type remains insufficiently studied but is considered the most aggressive (24). Studies evaluating the prognostic value of biomarkers in correlation with Lauren’s classification, which is widely used in clinical practice, have brought new knowledge and perspectives (9, 25). Studies of the correlation of combinations of several biological markers with clinical and pathological features taking into account the Lauren classification are also promising today (26, 27). Despite this, the prognostic significance of using Lauren’s classification and its combination with biomarkers remains unclear and relevant. It is necessary to continue the search for new prognostic biomarkers that will improve the assessment of relapse and individual prognosis.

Bibliometric analysis is a modern statistical tool for identifying development trends and research hot spots, for quantitative and qualitative assessment scientific papers in various fields (28). This study aims to provide a quantitative overview of the accumulated knowledge over a 29-year period on research dedicated to the prognostic value of gastric cancer biomarkers in relation to Lauren’s morphological classification, as well as to offer insights for future investigations.

In this study, we analyze the global scientific literature on evaluation of the prognostic importance of biomarkers by type of gastric cancer according to Lauren over 29 years. Our goal is to organize existing knowledge, identify key defining key countries, universities, authors, the most contributing journals, keywords and trending topics. This study is a kind of scientific navigator, helping to guide future research in this field.

## 2 Methods

### 2.1 Strategy for searching relevant publications in the Scopus database and inclusion and exclusion criteria

**Figure 1:**
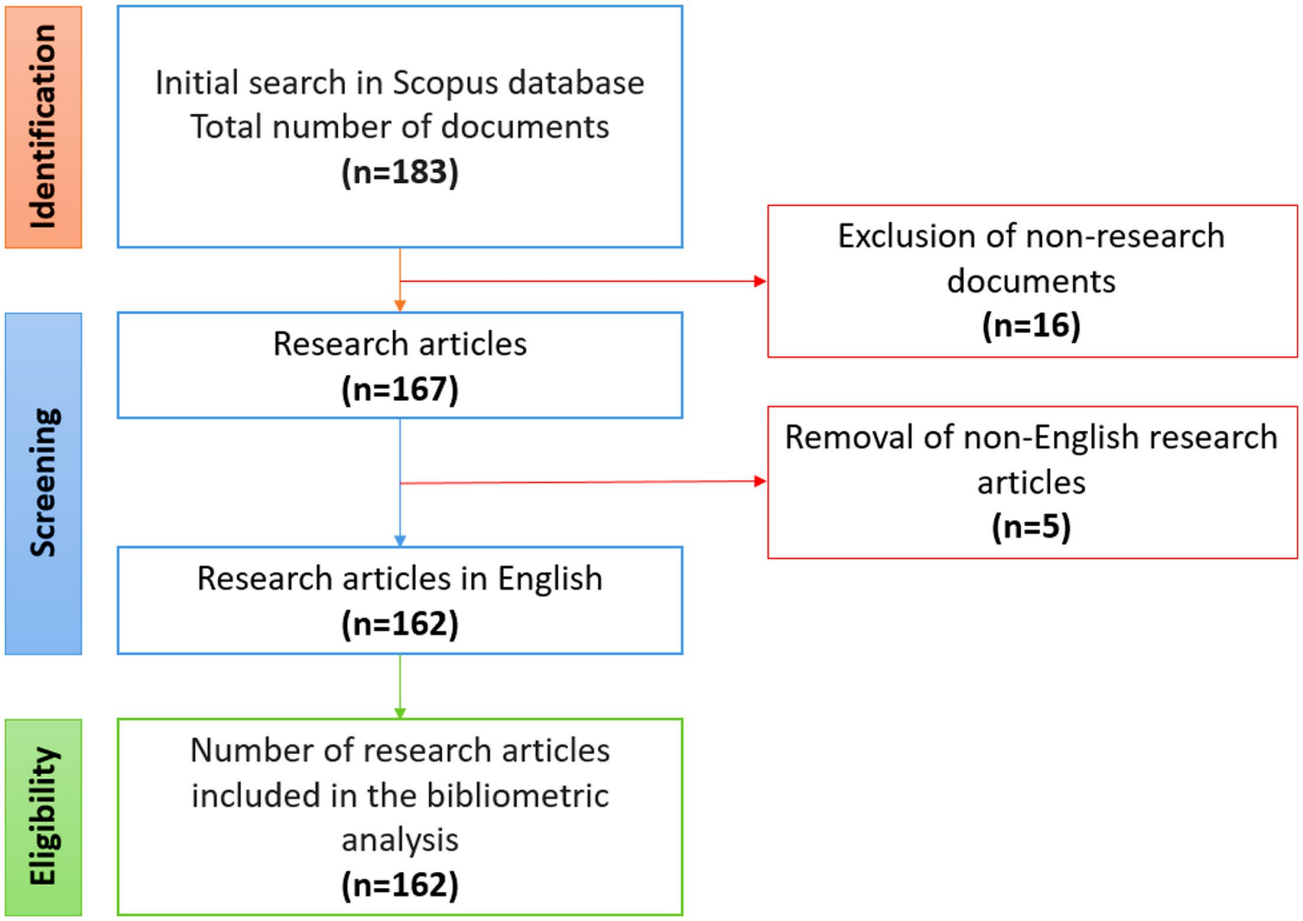
Flow chart of selection of research articles of biomarkers in gastric cancer depending on the morphological types according to the Lauren classification.

The search for scientific publications was conducted in the Scopus database in December 2024 using advanced search. Scopus is one of the largest abstract databases, covering a significant number of peer-reviewed scientific publications and widely used in bibliometric studies. To identify relevant keywords, we employed a combination of Boolean and wildcard search operators, which allowed us to significantly optimize the relevance of the sample (Table 1). Initially, 183 publications were found. After applying the inclusion and exclusion criteria, 21 articles were excluded. Only research articles in English were selected and extracted for analysis. A total of 162 research articles over a 29-year period were imported with all relevant metadata in BibTeX format. The data were analyzed using integrated development environment (IDE) RStudio. The steps of the complete search strategy are shown in detail in Figure 1.

**Table 1:** Queries used to search the Scopus database.

Code	Queries
#1	“Biomarker” OR “Marker, Biological” OR “Biological Marker” OR “Markers, Biological” OR “Biological Markers” OR “Biologic Markers” OR “Markers, Biologic” OR “Biologic Marker” OR “Marker, Biologic” OR “Markers, Clinical” OR “Clinical Marker” OR “Marker, Clinical” OR “Clinical Markers” OR “Surrogate Markers” OR “Marker, Surrogate” OR “Surrogate Marker” OR “Markers, Surrogate” OR “Surrogate Endpoints” OR “Endpoints, Surrogate” OR “Surrogate End Points” OR “End Points, Surrogate” OR “Surrogate Endpoint” OR “Endpoint, Surrogate” OR “Surrogate End Point” OR “End Point, Surrogate” OR “Markers, Immunologic” OR “Immune Marker” OR “Marker, Immune” OR “Immune Markers” OR “Markers, Immune” OR “Immunologic Marker” OR “Marker, Immunologic” OR “Immunologic Markers” OR “Markers, Laboratory” OR “Laboratory Marker” OR “Marker, Laboratory” OR “Laboratory Markers” OR “Serum Markers” OR “Marker, Serum” OR “Serum Marker” OR “Markers, Serum” OR “Viral Markers” OR “Viral Marker” OR “Marker, Viral” OR “Markers, Viral” OR “Biochemical Marker” OR “Marker, Biochemical” OR “Biochemical Markers” OR “Markers, Biochemical”
#2	“Neoplasm, Stomach” OR “Stomach Neoplasm” OR “Gastric Neoplasms” OR “Gastric Neoplasm” OR “Neoplasm, Gastric” OR “Neoplasms, Gastric” OR “Neoplasms, Stomach” OR “Cancer of Stomach” OR “Stomach Cancers” OR “Cancer of the Stomach” OR “Gastric Cancer” OR “Cancer, Gastric” OR “Cancers, Gastric” OR “Gastric Cancers” OR “Stomach Cancer” OR “Cancers, Stomach” OR “Cancer, Stomach” OR “Gastric Cancer, Familial Diffuse”
#3	“Prognoses” OR “Prognostic Factors” OR “Prognostic Factor” OR “Factor, Prognostic” OR “Factors, Prognostic”
#4	“Lauren Classification” OR “Lauren’s classification”
#5	#1 and #2 and #3 and #4

### 2.2 Bibliometric analysis

Bibliometric analysis and data visualization were performed using RStudio version 2024.12.0, a well-known statistical software that is open source and freely available for use, and the Biblioshiny package version 4.3.0.

## 3 Results

### 3.1 General characteristics of the output data of scientific publications

**Figure 2:**
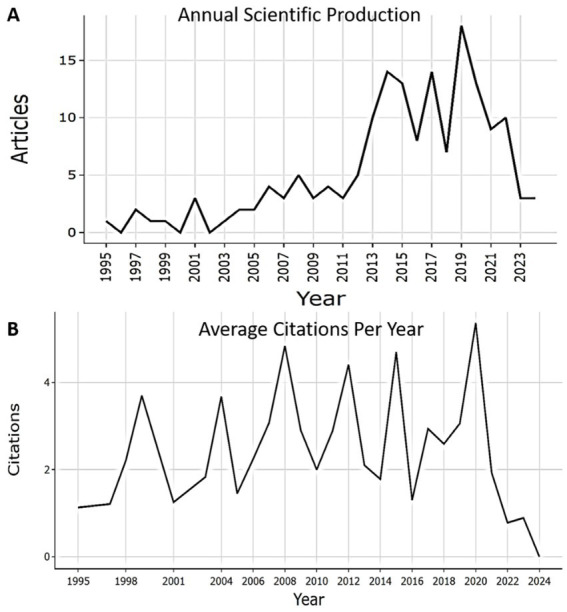
**(A)** Annual production of research articles from 1995 to 2024. **(B)** Average citations per year from 1995 to 2024.

The time interval of 162 articles retrieved from Scopus on the prognostic value of biomarkers in gastric cancer depending on histological types according to the Lauren classification covered 29 years (from 1995 to 2024), which demonstrates a fairly long period of interest in this topic among the scientific community. The average annual growth rate of publication activity was 3.86%, indicating a moderate, but at the same time stable development of interest in this area (Figure 2). The demand for scientific papers on this topic is evidenced by the high average document age of 10.1 and the average number of citations per document of 30.4. The full bibliometric breakdown of the search results is shown in Table 2.

**Table 2:** General description of data from scientific publications devoted to the study of the prognostic significance of biological markers of gastric cancer depending on the Lauren subtypes.

Description	Results
Main information about data	
Time span	1995:2024
Sources (journals, books, etc.)	96
Documents	162
Annual growth rate %	3.86
Document average age	10.1
Average citations per doc	30.4
Document contents	
Keywords plus (ID)	1,629
Author’s keywords (DE)	350
Authors	
Authors	915
Authors of single-authored docs	–
Authors collaboration	
Single-authored docs	–
Co-authors per doc	7.94
International co-authorships %	15.43

The ratio of 915 authors to 162 articles (total number of documents), approximately 8 authors per article and the absence of single authors demonstrates a high level of collectivity in the study of this area. At the same time, the share of international co-authorship was 15.43%, indicating a moderate level of involvement in global scientific collaborations (Table 2). The richest years in publications devoted to the definition prognostic value of biomarkers in different histological types of gastric cancer according to Lauren are 2019 (18 articles), 2014 (14 articles) and 2017 (14 articles). From 2019 to 2021, there is a sharp decrease in publications by almost half (from 18 publications to 9 publications) with a subsequent decrease (Figure 2A). In 2008, the maximum Mean TC per year was recorded (4.84) with 5 articles. In 2020, a second peak was observed (5.37) (Figure 2B).

### 3.2 Country productivity and global cooperation

**Figure 3:**
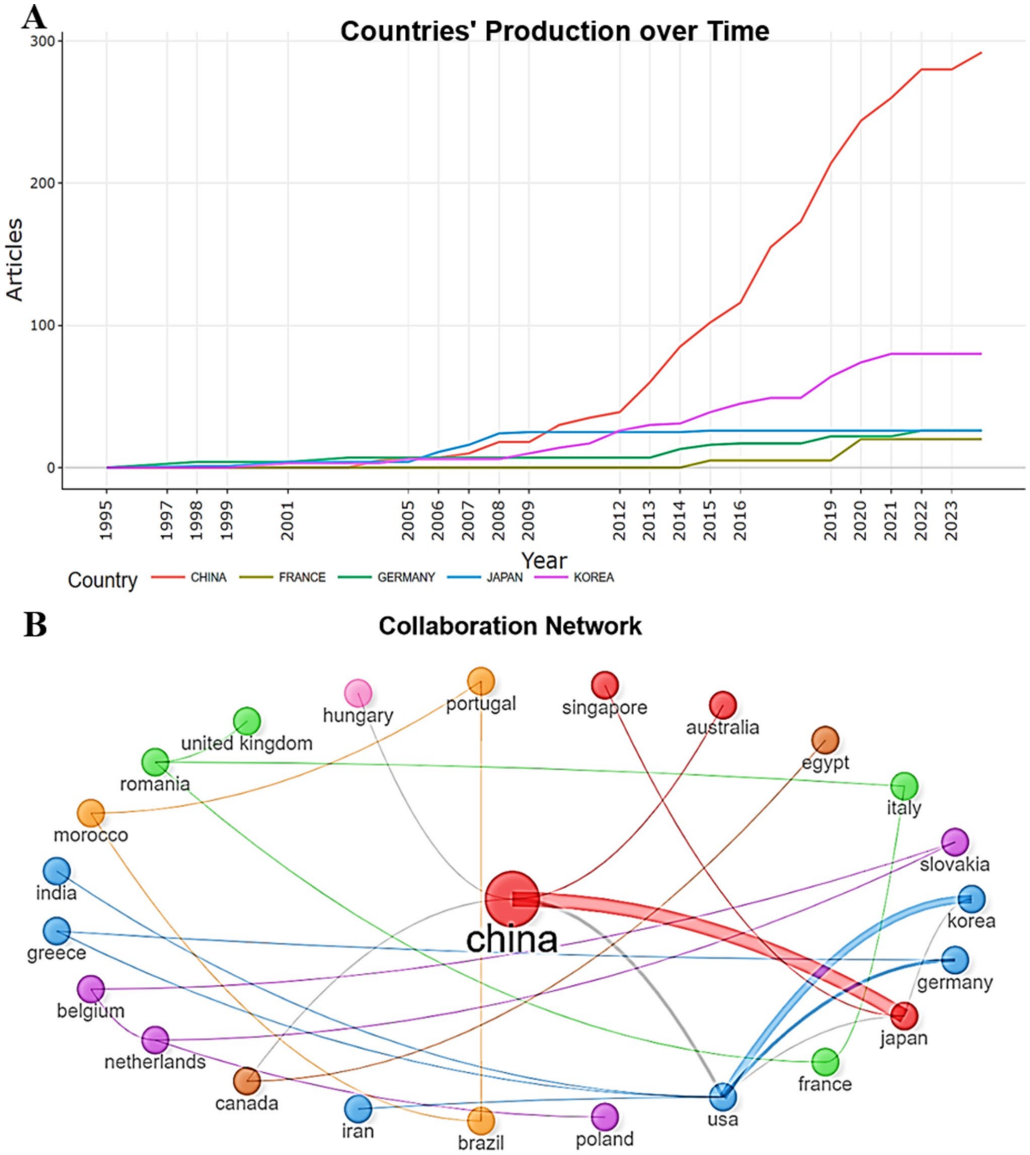
**(A)** Distribution of the number of publications by top 5 countries by year (1995–2024). **(B)** Visualization of the network of countries’ collaboration in scientific papers related to assessment of the prognostic significance of biomarkers depending on the type of gastric cancer according to Lauren (1995–2024). In visualizing the country collaboration network, research articles were divided into six distinct color clusters. This classification used the Star layout using the edge betweenness clustering algorithm, including 50 nodes, removing isolated nodes, a minimum threshold of two edges, and using 1,000 labels.

To evaluate the scientific potential of countries and regions, we studied the publication and citation metrics. The analysis of country productivity helps those interested in this area to better understand the research contribution of leading countries, better navigate and make decisions about participating in studies related to the study of the prognostic capabilities of gastric biomarkers depending on the Lauren morphological classification. A total of 27 countries were identified that published scientific papers in this field. The undisputed leader in annual publications from 2010 (30 documents) to 2024 (292 documents) is China, followed by Korea (80 documents), Germany (26 documents), which emphasizes the significant contribution of the Asian and European research communities to the development of this field (Figure 3A). The indicators show that these countries have vast experience and wealth of knowledge, high scientific potential in research in this area.

International collaboration in solving common problems in studying prognostic biomarkers of gastric cancer is one of the integral components for effective multi-vector development of this area. The network of global cooperation of countries in scientific works devoted to this area is presented in Figure 3B. The top three closely cooperating countries are China, the USA and Japan. The visualization demonstrates the strong ties of China and the USA in the network of scientific cooperation, reflecting their key position as an intermediary. Countries such as the Netherlands and Romania have a high level of information availability in the network. Brazil, Morocco and Portugal have high connectivity within their clusters.

### 3.3 Analysis of authors’ productivity and their collaboration

**Figure 4:**
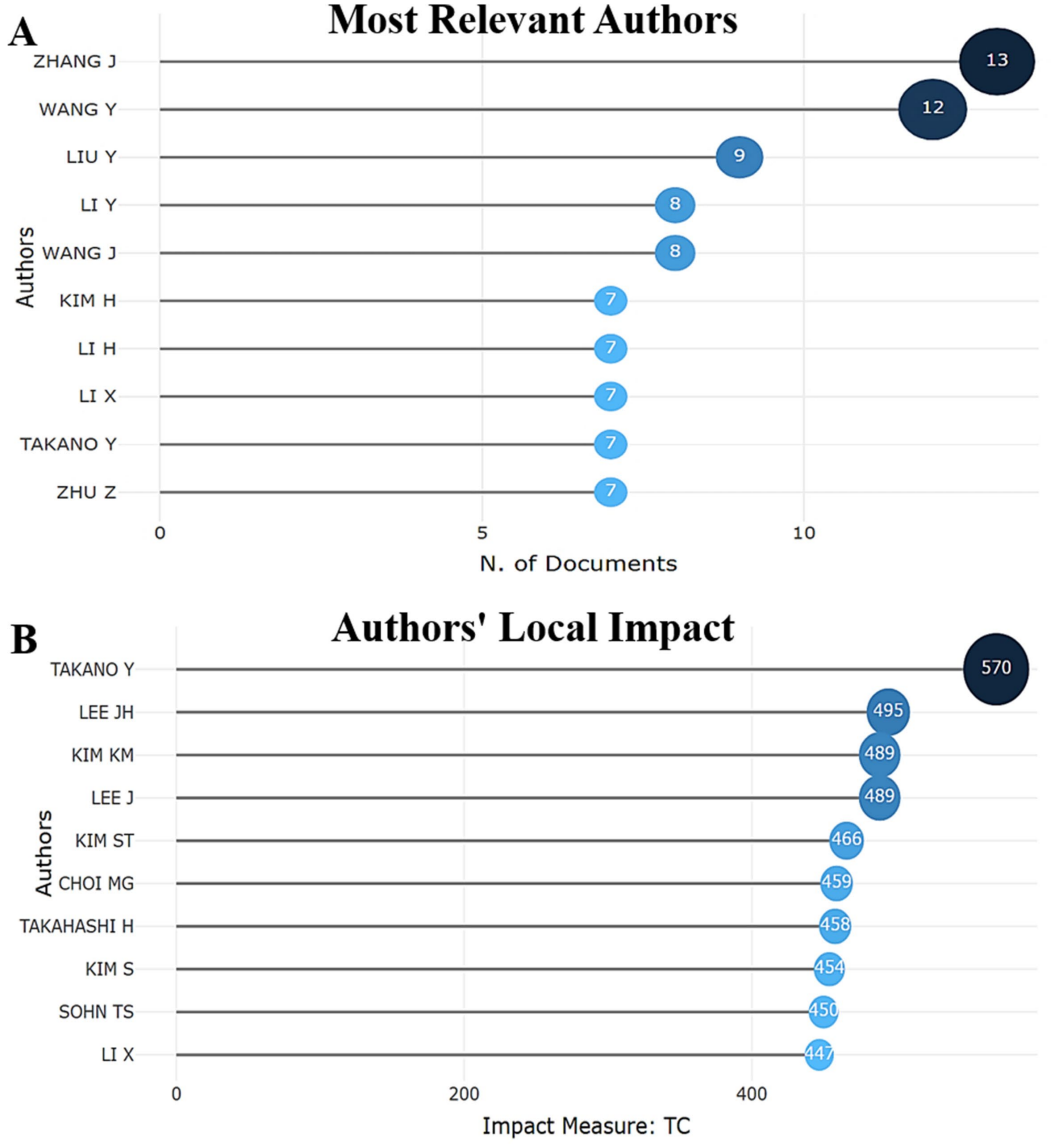
**(A)** Top 10 most prolific authors who have published scientific papers on prognostic assessment of biomarkers depending on the Lauren histological classification in gastric cancer. **(B)** Top 10 leaders according to authors’ local impact in this topic.

The analysis of the authors’ scientific productivity and their interactions helps to identify the key players and their influence, as well as the degree of their involvement in international scientific collaboration. Zhang J. ranks first by the number of articles (13 documents), he is also one of the most collaborating authors. In second place is Wang Y. with 12 documents, who made a significant individual contribution to the study of this topic. Liu Y. is in third place with 9 studies, where he was the lead investigator (Figure 4A). It is noteworthy that Takano (570 TC) is the undisputed leader, confirming his status as a creator of benchmark works. Lee JH. (495 TC) and Kim KM. (489 TC) are new key players (absent from previous productivity tops Figure 4A), whose works resonate in the community (Figure 4B).

**Figure 5:**
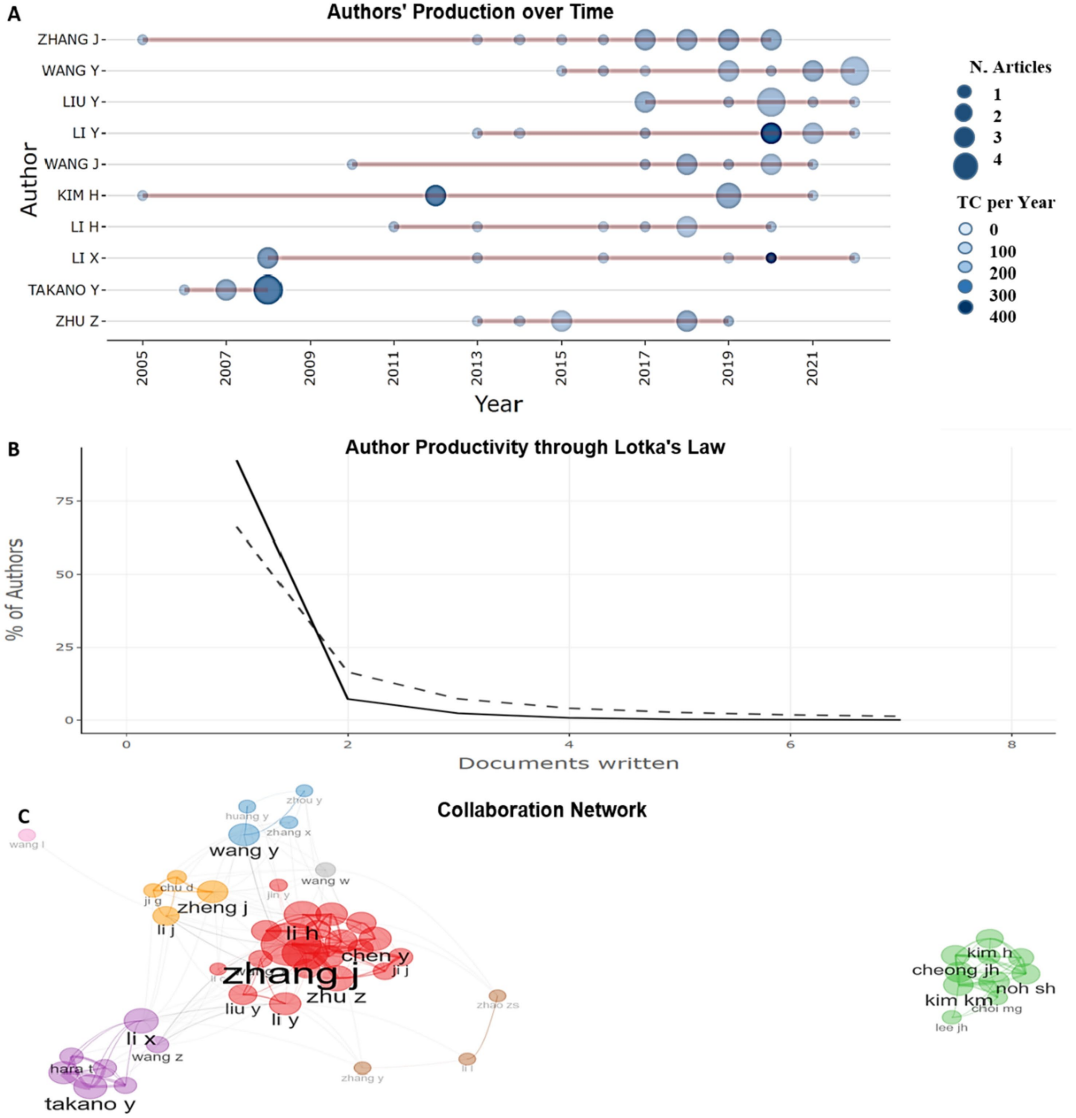
**(A)** Timeline of production of 10 significant contributors to the study the importance of biomarkers in predicting the course of gastric cancer by Lauren types (2005–2022). **(B)** Analysis of authors’ productivity using Lotka’s law. **(C)** Visualization of collaboration between authors. The color of the dots indicates their belonging to the author cluster group, the thickness of the line reflects the closeness of the collaboration.

When analyzing author production over time (2005–2022), we found an uneven distribution of productivity among top authors and by year (Figure 5A). The most productive and influential are Takano Y. (2008): (4 publications, 376 citations, TCpY = 20.889), Liu Y. (2020): 4 publications, 30 citations, TCpY = 5, Wang Y. (2022): 4 publications, 14 citations, TCpY = 3.5. The citation impact evolution (TCpY) analysis identified 3 authors with the highest citation count: Li Y. (2020): 2 publications, 176 citations; Li X. (2020): 1 publication, 169 citations, TCpY = 28.167, TCpY = 29.333; Takano Y. (2008): 4 publications, 376 citations, TCpY = 20.889. The analysis showed an increase in citation count for new documents, while the level remains high for old ones. The low level of publication activity of authors occurred in 2005–2012, TCpY varied within 1–2. In the period 2013–2017, an increase in activity was observed, authors with higher TCpY (up to 8.444) appeared. In 2018–2022. The maximum growth in citation occurred, some publications reached TCpY> 25. The publication activity of authors tends to increase, which may indicate an expansion of the scientific community in this topic.

The analysis of authors’ productivity according to Lotka’s law showed that the majority of authors (730/79.8%) wrote only one document, 107 authors (11.7%) wrote two documents. The number of authors who wrote three or more documents is significantly reduced (from 30 to 1). The presence of one author with 9, 12 and 13 documents indicates the rarity of highly productive researchers, a limited number of leading researchers or experts in the field. About 90% of authors have 1–2 publications, which indicates a high proportion of occasional authors, or this is an indication of a high involvement of young or new researchers. Only about 1% of authors are highly productive scientists with six or more publications (Figure 5B).

The research collaboration network was divided into 8 clusters, each reflecting groups of closely interacting authors (Figure 5C). Zhang J and Li X are key figures, ensuring interactions between different research groups. Zhang J acts as a “bridge” between different authors from other groups. Scientists such as Kim H, Lee JH and Kim KM closely interact within their group. Thus, the collaboration network demonstrates a complex hierarchy of scientific interactions, where some researchers play the role of liaisons, while others focus on internal interactions within their groups.

### 3.4 Analysis of publications and the most significant scientific works

Over 29 years, ranking of the most popular and most cited studies on study of the significance of biomarkers in predicting the course of gastric cancer depending on the Lauren classification leads the research Park et al. «Phase III Trial to Compare Adjuvant Chemotherapy With Capecitabine and Cisplatin Versus Concurrent Chemoradiotherapy in Gastric Cancer: Final Report of the Adjuvant Chemoradiotherapy in Stomach Tumors Trial, Including Survival and Subset Analyses” (381TC), in second place is the document “Overexpression of GRP78 and GRP94 are markers for aggressive behavior and poor prognosis in gastric carcinomas» Zheng et al. (173TC), and the top three is rounded out by an article by Chinese scientists Peng et al. “CirCUL2 regulates gastric cancer malignant transformation and cisplatin resistance by modulating autophagy activation via miR-142-3p/ROCK2” (Table 3).

**Table 3:** Ranking of the 10 most cited research articles on assessment prognostic significance of biomarkers in gastric cancer of different types according to Lauren.

Ranking	Study references	Title of the document	Journal name	Total citations	DOI
1	PARK SH, 2015	Phase III Trial to Compare Adjuvant Chemotherapy With Capecitabine and Cisplatin Versus Concurrent Chemoradiotherapy in Gastric Cancer: Final Report of the Adjuvant Chemoradiotherapy in Stomach Tumors Trial, Including Survival and Subset Analyses	Journal of Clinical Oncology	381	10.1200/JCO.2014.58.3930
2	ZHENG H, 2008	Overexpression of GRP78 and GRP94 are markers for aggressive behavior and poor prognosis in gastric carcinomas	Human Pathology	173	10.1016/j.humpath.2007.11.009
3	PENG L, 2020	CircCUL2 regulates gastric cancer malignant transformation and cisplatin resistance by modulating autophagy activation via miR-142-3p/ROCK2	Molecular Cancer	169	10.1186/s12943-020-01270-x
4	AN JY, 2012	Microsatellite instability in sporadic gastric cancer: its prognostic role and guidance for 5-FU based chemotherapy after R0 resection	International Journal of Cancer	137	10.1002/ijc.26399
5	ZHENG H, 2007	Pathobiological characteristics of intestinal and diffuse-type gastric carcinoma in Japan: an immunostaining study on the tissue microarray	Journal of Clinical Pathology (JCP)	129	10.1136/jcp.2006.038778
6	ZHENG HC, 2008	Mixed-type gastric carcinomas exhibit more aggressive features and indicate the histogenesis of carcinomas	Virchows Archive	112	10.1007/s00428-007-0572-7
7	DRIESSEN A, 2006	Expression of Carbonic Anhydrase IX (CA IX), a Hypoxia-Related Protein, Rather Than Vascular-Endothelial Growth Factor (VEGF), a Pro-Angiogenic Factor, Correlates With an Extremely Poor Prognosis in Esophageal and Gastric Adenocarcinomas	Annals of Surgery	111	10.1097/01.sla.0000201452.09591.f3
8	ZHANG H, 2015	Infiltration of diametrically polarized macrophages predicts overall survival of patients with gastric cancer after surgical resection	Gastric Cancer	109	10.1007/s10120-014-0422-7
9	CHEN CN, 2004	The significance of placenta growth factor in angiogenesis and clinical outcome of human gastric cancer	Cancer Letters	108	10.1016/j.canlet.2004.05.020
10	POLKOWSKI W, 1999	Prognostic Value of Laurén Classification and c-erbB-2 Oncogene Overexpression in Adenocarcinoma of the Esophagus and Gastroesophageal Junction	Annals of Surgical Oncology	100	10.1007/s10434-999-0290-2

### 3.5 Analysis of the most prolific journals

**Figure 6:**
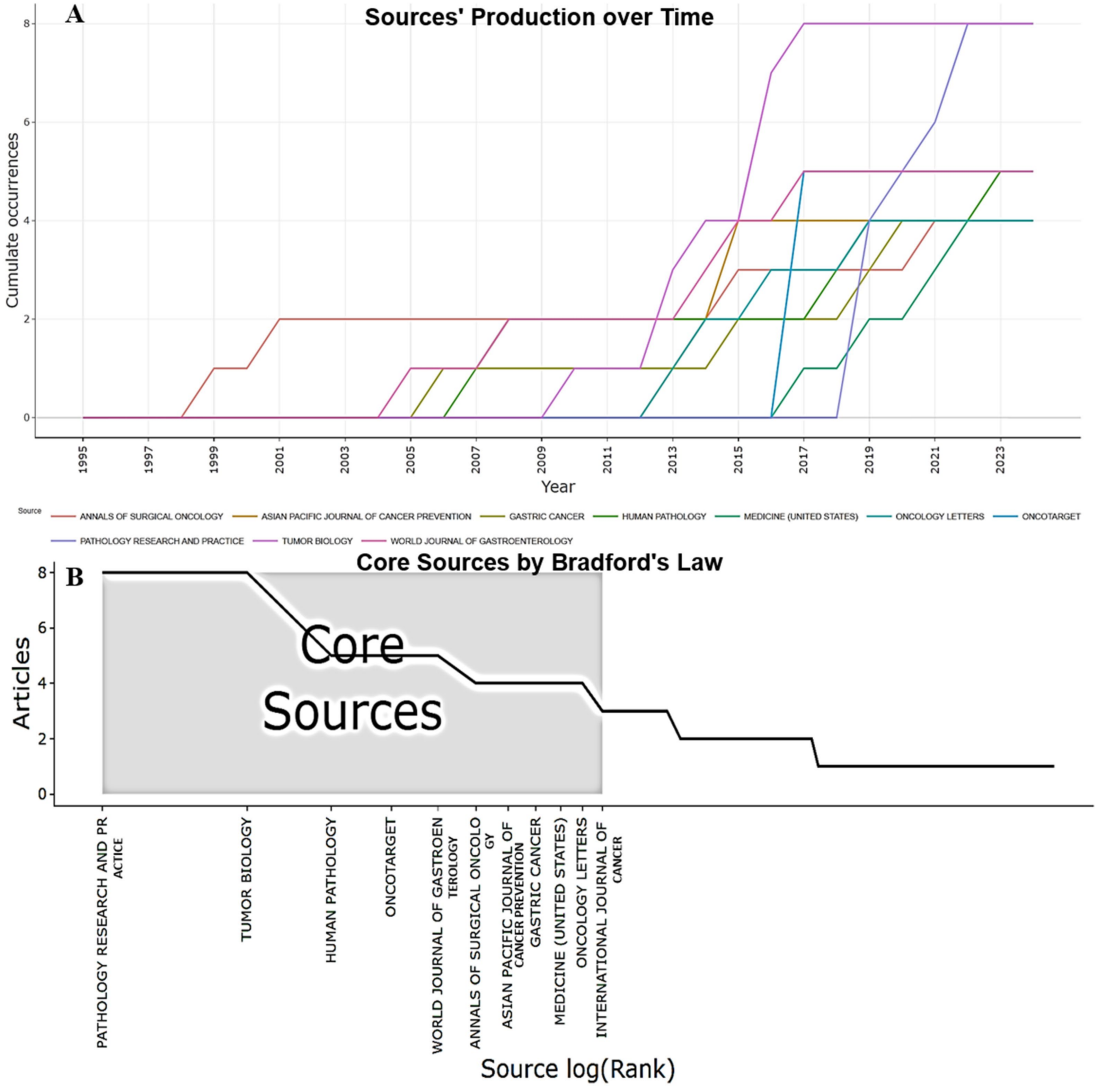
**(A)** Production of the top 10 prolific journals over 29 years. The figure shows the cumulative productivity of journals, with journals shown as colored lines, each line representing data for a specific journal. **(B)** Distribution map of the top journals using Bradford’s law.

Bibliometric analysis of journals allows us to determine the top journals publishing articles on the role of biomarkers in determining the prognosis of gastric cancer depending on the histological type according to Lauren. Analysis of journal output in the period from 1995 to 2024 shows the stability of high rates in a number of leading scientific journals, which confirms the relevance of the topic and the increase in the number of publications in this area. At the initial stage from 1995 to 2004, publication activity was extremely low, there were practically no articles. However, starting in 2005, there has been a gradual increase in publications, especially in the World Journal of Gastroenterology and Annals of Surgical Oncology. Since 2010, a steady increase in publication activity has begun, especially in journals such as Tumor Biology (8 documents in total) and Human Pathology (5 publications in total). In 2017, a sharp increase in publication activity was recorded in “Tumor Biology,” which reached 8 publications, as well as a significant increase in activity in “Oncotarget” (5 publications). This trend continued in subsequent years, demonstrating a consistently high interest in the topic. Since 2019, “Pathology Research and Practice” began to actively increase publications, reaching its peak in 2022–2024 (a total of 8 documents). Similar dynamics are observed in “Oncology Letters,” which confirms the increasing role of these publications in covering the prognostic significance of biomarkers in gastric cancer. In 2020–2024, it can be noted that a number of journals (e.g., “Medicine (United States),” “Gastric Cancer”) stabilized their publication activity at the level of 4–5 articles per year, which indicates an established research trend (Figure 6A).

Bradford’s Law identifies the most authoritative sources in a field of research the importance of biomarkers in predicting the outcome of gastric cancer by Lauren type. This analysis shows the concentration of scientific publications in certain highly cited journals. Eleven publications were included in the list of prestigious journals for publication with the highest publication frequency. The journals Pathology Research and Practice and Tumor Biology occupy the first positions in the ranking of the most popular journals in the scientific community, they have the largest number of publications (8 articles each). Among the most scientifically active journals, oncology and pathology journals predominate, such as Oncotarget, Human Pathology, Annals of Surgical Oncology and International Journal of Cancer. Specialized journals on gastroenterology are also presented: World Journal of Gastroenterology, Gastric Cancer (Figure 6B). Journals with high citation rates continue to set scientific trends in research related to the study of the significance of biomarkers in predicting the course of gastric cancer depending on the Lauren classification, and new publications contribute to the development of highly specialized areas, which expands the horizons of research and strengthens interdisciplinary scientific connections.

**Figure 7:**
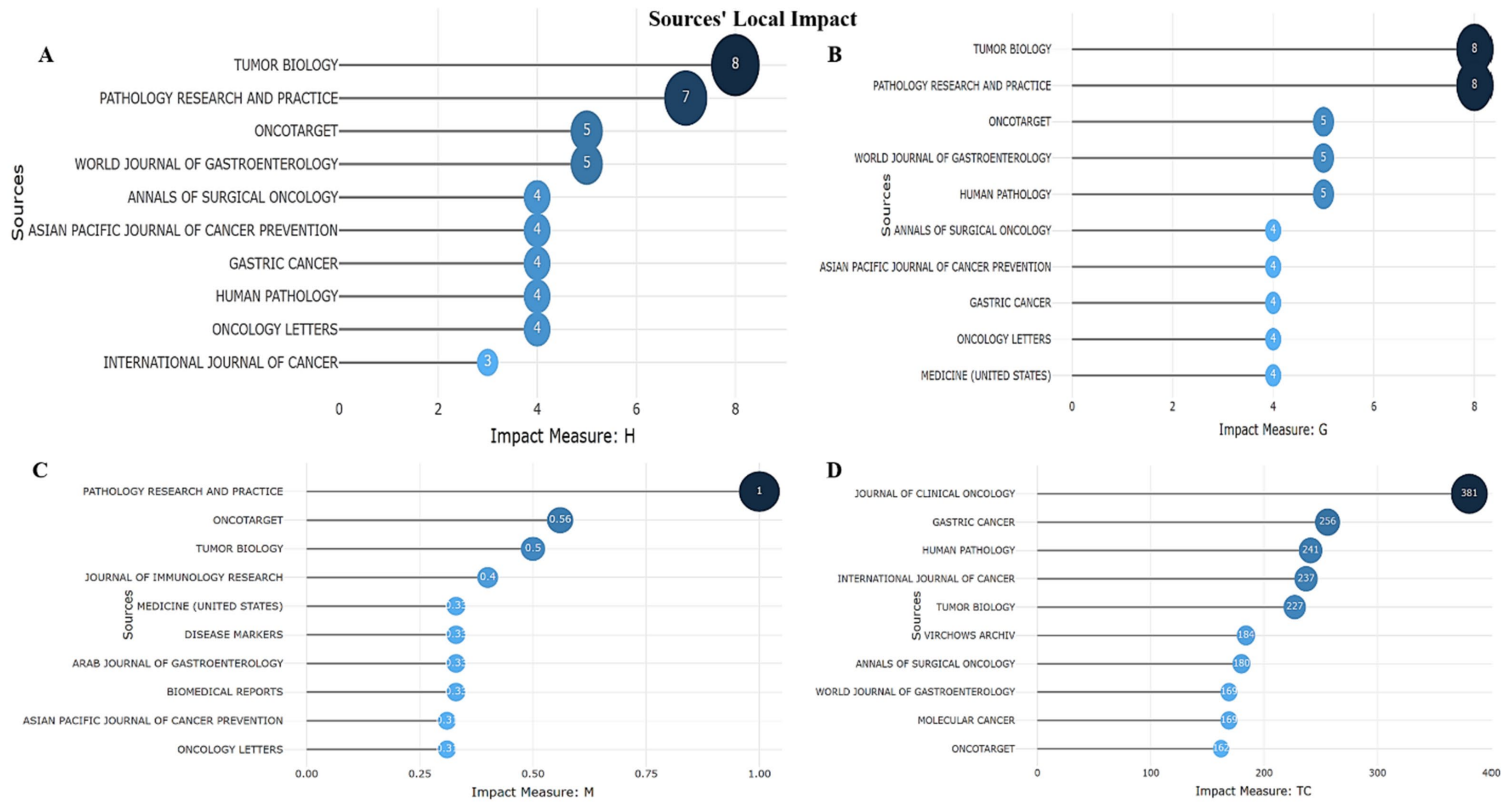
Top 10 journals with local impact **(A)** by h_index. **(B)** by g_index. **(C)** by m_index. **(D)** by total citations.

The highest number of citations and the highest Hirsch index were found in the following journals: “Tumor Biology” (h_index-8, TC-227), “Pathology Research and Practice” (h_index-7, TC-116), and “Oncotarget” (h_index-5, TC-162). This indicates stable citation of works published in these scientific journals. The journal with the most stable influence (h_index) was “Tumor Biology” (Figure 7A). “Human Pathology” demonstrates a g-index (5) and TC = 241, indicating stable demand for publications (Figure 7B). “Pathology Research and Practice” is distinguished by a high level of research relevance. Its m-index is the highest among all sources, indicating the rapidly growing importance of publications (Figure 7C). “Journal of Clinical Oncology” (PY_start = 2015) has only an h-index of 1, but TC = 381, indicating the high significance of its publications for the scientific community (Figure 7D).

### 3.6 Analysis of the contribution of institutions and their cooperation

Studying the research contributions of institutions allows for better orientation and participation in research prognostic assessment of biomarkers depending on the Lauren histological classification in gastric cancer. Table 4 presents data on the 10 leading institutions that have made a significant contribution to the development of this area.

**Table 4:** The top 10 affiliations that published research articles.

Rank	Affiliation	Articles
1	Sungkyunkwan University School of Medicine	13
2	Fudan University	12
3	Yonsei University College of Medicine	11
4	Fourth Military Medical University	10
5	Shanghai Jiao Tong University School of Medicine	9
6	Zhejiang Provincial People’s Hospital	9
7	Chang Gung University	8
8	University of Toyama	8
9	Catholic University of Korea	6
10	China Medical University	6

Among the institutions with the highest publication rate in this field, Sungkyunkwan University School of Medicine ranks first with 13 articles, followed by Fudan University (12 articles) in second place and Yonsei University College of Medicine (11 articles) in third place (Table 4).

**Figure 8:**
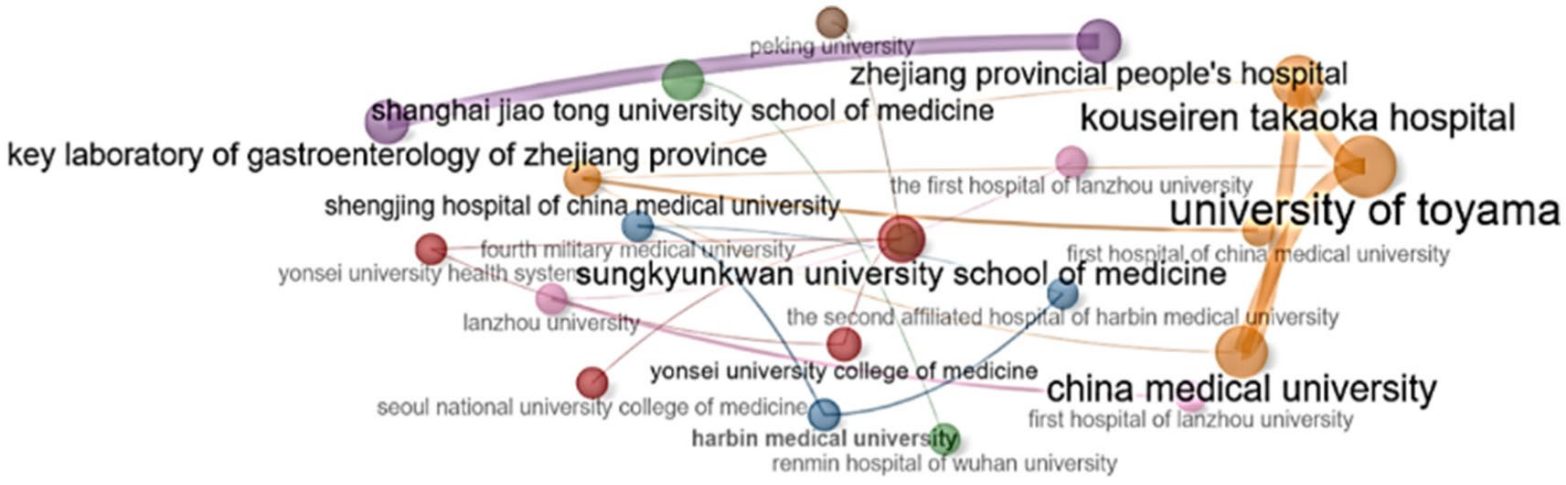
Collaboration between scientific institutions. The institutions were grouped into seven separate clusters.

The analysis of university collaborations showed the importance of strong regional collaboration centers in the medical and academic environment, and also identified organizations that play a critical role in the dissemination of knowledge and scientific connections. Figure 8 shows that the absolute leaders in the level of collaboration are Zhejiang Provincial People’s Hospital, Peking University, Shanghai Jiao Tong University School of Medicine and Renmin Hospital of Wuhan University, playing a key role in scientific connections. They act as interaction centers, providing communication between other institutions. Special attention should be paid to Shengjing Hospital of China Medical University, which plays an intermediary role in the academic environment. Lanzhou University also stands out for its significant influence on the structure of the collaboration network. Sungkyunkwan University School of Medicine, Yonsei University College of Medicine, Seoul National University College of Medicine and Yonsei University Health System play significant roles in the collaboration network (Figure 8).

### 3.7 Analysis of author keywords and trending topics

**Figure 9:**
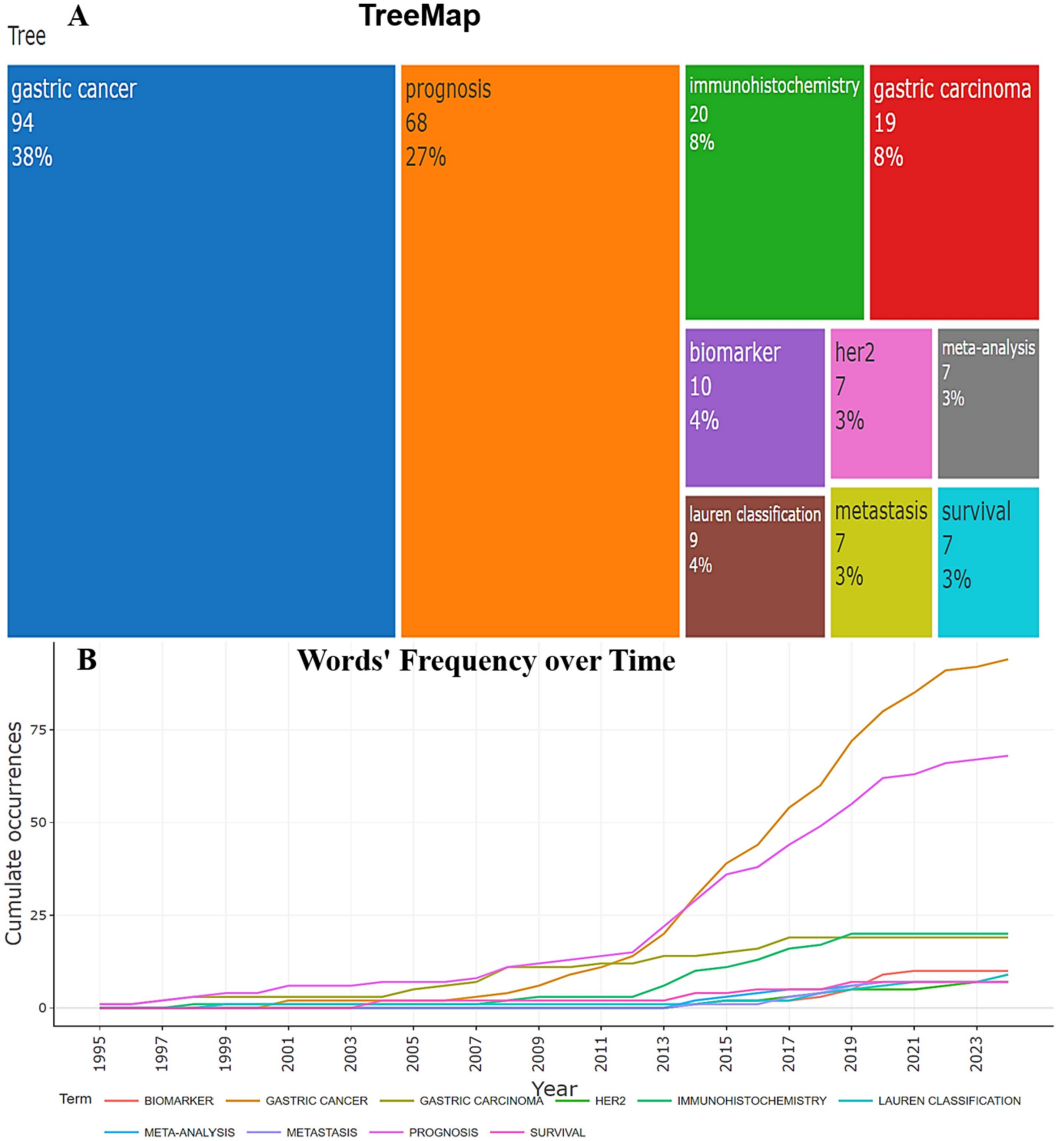
**(A)** Tree map of the top 10 most frequently occurring keywords of the author. The color of the rectangle represents the specific keyword of the author. The larger the area of the rectangle, the more frequently the author’s keyword appears. **(B)** Scatter plot representing the frequency of use of the author’s 10 important keywords in studies prognostic assessment of biomarkers in gastric cancer according to the Lauren classification (1995–2024).

A total of 350 author keywords were included in this analysis (Table 2). Tree map Figure 9A shows, that the leading position is occupied by “gastric cancer” (94 mentions), which confirms the relevance and high level of scientific attention to this topic. “Prognosis” (68 mentions) is the second significant word, which indicates the important role of predicting disease outcomes. Particular attention is paid to diagnostic methods and the identification of biomarkers, the traditional histological classification of gastric cancer according to Lauren remains a relevant tool, as evidenced by the frequency of occurrence of the keywords “Immunohistochemistry” (20 mentions) and “Biomarker” (10 mentions), “Lauren classification” (9 mentions). Over the past 29 years, there has been a significant increase in interest in the study of stomach cancer and its prognosis. Analysis of the frequency of use of key author terms in publications allows us to identify the main areas of research. Growing interest in the topic of “gastric cancer” - since 2010, the number of publications on this topic has been steadily increasing, reaching 94 mentions in 2024. This indicates a high relevance of studying stomach cancer among cancers of other organs. Disease prediction (“prognosis”) is also an important area of research, demonstrating a steady increase from 1 publication in 1995 to 68 in 2024. “immunohistochemistry” and “biomarker” gained popularity after 2015, which is most likely due to the significant development of molecular diagnostic methods. Before 2005, research was limited, with a small number of publications. 2010–2015—a sharp increase in interest in the topic of stomach cancer and related factors, which is associated with the development of personalized medicine. Since 2016, there has been a steady increase in the number of publications, especially in the field of prognosis and molecular markers (Figure 9B). The presented data reflect the most important trends in modern oncology, emphasizing the importance of diagnostic and prognostic methods for gastric cancer.

**Figure 10:**
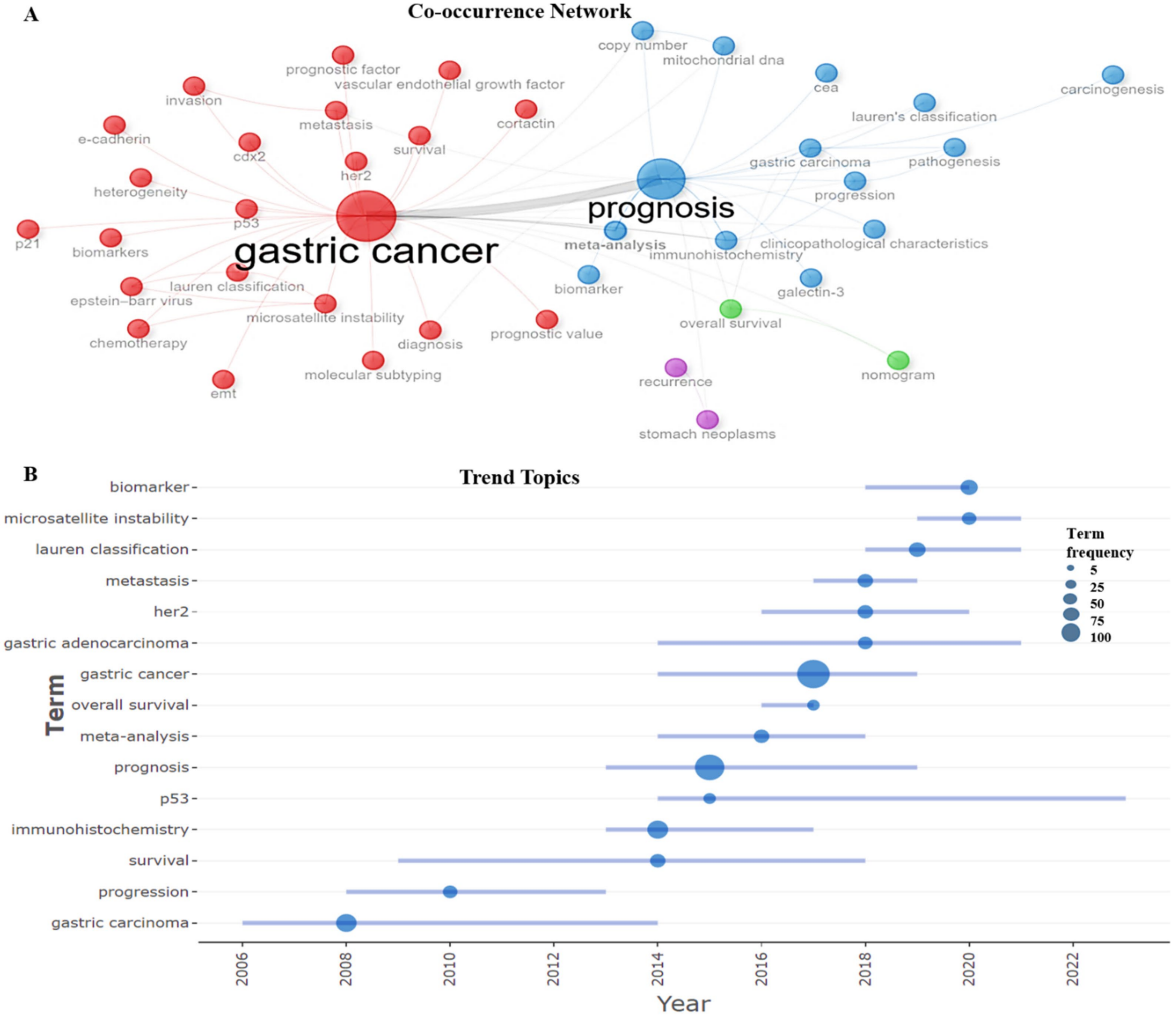
**(A)** Co-occurrence analysis of author keywords using Biblioshiny. Each node represents an author keyword, and its size reflects its frequency of occurrence. The larger the node, the more frequently the author keyword occurs. The line reflects the co-occurrence relationship between author keywords, and the color indicates group membership. **(B)** Analysis of research topic trends by author keywords. Each ball indicates the peak frequency of use, and the line indicates the years in which the author keywords were used.

The study of the author keyword co-occurrence network reveals significant trends and relationships between different aspects of the areas in the field. Figure 10A illustrates that the keyword “Prognosis” as well as “Gastric cancer” also ranks high, indicating its importance in the study of disease outcomes. “HER2,” “metastasis,” “microsatellite instability,” “p53,” “vascular endothelial growth factor,” “Lauren classification” are associated with “Gastric cancer” in this cluster. This indicates active study of molecular mechanisms and possible therapeutic targets. The cluster consisting of the keywords “prognosis,” “gastric carcinoma,” “progression,” “clinicopathological characteristics,” “Lauren’s classification” demonstrates the interest of researchers in disease prognosis and treatment individualization, reflecting two main research areas: molecular biological aspects and clinical pathological characteristics of the disease. Thus, the analysis of scientific terms shows that research in the field of gastric cancer focuses on both molecular mechanisms and clinical predictors of the disease, which is important for improving diagnostics and personalized treatment.

Analysis of trending topics according to the author’s keywords shows how the priority areas of gastric cancer research have changed over time (Figure 10B). The terms “gastric carcinoma” and “progression” began to be actively used in 2006–2008, reflecting the initial stages of studying disease progression. The most actively used method is “immunohistochemistry,” with a sharp increase in the number of publications in 2013–2017. In 2014, there was a surge in publications related to p53, meta-analysis, gastric adenocarcinoma and gastric cancer, which may indicate an increase in interest in the molecular genetic aspects of gastric cancer diagnostics.

The term prognosis remains dominant, with a high frequency of occurrence in the scientific works of 2013–2019. High interest in metastasis with a peak in publication activity in 2017–2019 confirms the significance of this problem. Since 2009, attention has been growing to “survival,” which indicates the focus of the studies on the prognosis of the course of gastric cancer in patients. Starting from 2018, the key topics were “biomarker,” “her2” (2016–2020), “microsatellite instability,” indicating the increasing role of personalized medicine in the treatment of gastric cancer. The topics of “gastric adenocarcinoma” and “Lauren classification” (with a peak number of publications in 2019) have remained relevant in recent years, emphasizing the active use of this classification of gastric cancer in modern scientific research. Thus, the presented data demonstrate how the priorities of scientific research have changed, emphasizing the role of new technologies in oncology.

## 4 Discussion

Using a bibliometric analysis of global publications from 1995 to 2024, we obtained a comprehensive and deep understanding of the current research situation in the field of study the role of biomarkers in the prognosis of gastric cancer taking into account the Lauren classification. In recent decades, the prognosis of gastric cancer remains unfavorable, so more effective prognostic markers are needed (12, 15). The analysis showed that studies concerning the prognostic value biomarkers with classification Lauren in the prognosis of gastric cancer, attracted the attention of many researchers (915authors) (Table 2). In general, the total volume of publications over 29 years has gradually increased, reaching its peak of 18 articles (2019). From 2019 to 2021 the number of publications sharply decreased by almost half (from 18 documents to 9 documents), after which a gradual decline continued (Figure 2A). During this period, global scientific activity may have slowed down due to restrictions related to the COVID-19 pandemic, which affected the number of publications in various fields of medicine. However, citation analysis shows that the Lauren classification remains an influential field. After 2019, the average number of citations per article per year (MeanTCperYear) reached 5.37 (2020), which is higher than many previous years. The decline in publications after 2019 is due to global factors (COVID-19), rather than a decrease in scientific interest (Figure 2B). The search for accurate diagnostic and prognostic biological markers for gastric cancer is a constant focus of research, as evidenced by the average document age of 10.1, the average number of citations per document of 30.4.

The leader in the number of works on this topic is China, the number of publications and total citations of which occupies the lion’s share of the total number (292 publications). In second place is South Korea, with the number of publications more than 3.5 times lower than China (80 articles). High productivity of these countries explained by the burden placed on them by stomach cancer and how financing as a response to epidemiology (2, 3, 29). A significant proportion of funding for gastric cancer research in these countries came from government grants, as evidenced by the strong correlation between the number of publications and R&D expenditure (30, 31). The contribution of European countries remains limited, despite the activity of individual research groups. Germany is not among the countries with the highest rates of stomach cancer in the world, but with 26 publications it is in third place (Figure 3A). Ivey et al. reported on the high incidence of diffuse subtype gastric cancer according to the Lauren classification in young women in European countries (24). China, USA and Japan actively collaborate in the study of biomarkers and their prognostic significance in gastric cancer, combining resources, data and expertise to achieve more accurate and valid results (Figure 3B). The combination of high incidence in countries where universities are located (3), significant investments, advanced technologies and active international cooperation allowed the universities Sungkyunkwan University School of Medicine (13 articles), Fudan University (12 publications), Yonsei University College of Medicine (11 articles) to become leaders in the number of publications (Table 4).

Among the researchers, Zhang J. stands out as the most productive, influential and most collaborative, with 385 citations to 13 papers. Wang Y. (12 papers) and Liu Y. (9 papers) are the second and third significant contributors, respectively (Figure 4A). Takano (TC = 570) represents the phenomenon of the “eternal landmark.” His 2008 concepts remain the methodological foundation of modern research (the 2008 article (TCpY = 20.89) is cited 4.7 times more often than the average article in the discipline) (Figure 4B). Clinicians should re-examine his early works in the context of new clinical paradigms. Ignoring these publications leads to methodological errors in interventional studies. Particularly relevant for the development of diagnostic algorithms. Li Y. represents an emerging leader in the field: his work (2020–2022) has become a catalyst for research in the field. The unique combination of methodological rigor (high fractional contribution) and clinical focus (explosive growth in citations) makes his publications a must-monitor for practitioners (record annual impact: TCpY = 29.33 (2020)—the maximum indicator, exponential growth: TCpY increase from 1.69 (2013) to 29.33 (2020)—a 17.3-fold increase in 7 years, significant citations (TC = 287)). Lee JH.—the main “surprise” of the analysis: his rare publications generate citation tsunamis (TC = 495 for only 6 articles, each publication is significant) (Figure 5A). Thematic analysis shows a focus on issues critical to clinical practice: optimization of therapeutic protocols and predictive diagnostics. Researchers should monitor his new works as indicators of emerging trends. Clinical value: reducing the time of implementation of innovations into practice.

The highest interest in the importance of biomarkers in predicting the course of gastric cancer by type Lauren was noted in 2020 (5.4 Mean TC per year) (Figure 2B). The most cited document is “Phase III Trial to Compare Adjuvant Chemotherapy With Capecitabine and Cisplatin Versus Concurrent Chemoradiotherapy in Gastric Cancer: Final Report of the Adjuvant Chemoradiotherapy in Stomach Tumors Trial, Including Survival and Subset Analyses” Park et al. published in the scientific journal “Journal of Clinical Oncology,” which was cited a record 381 times. This study investigated whether the addition of radiotherapy to adjuvant chemotherapy has a beneficial effect on disease-free survival (DFS) in patients with gastric cancer (GC) who underwent D2 resection. The benefits of DFS were analyzed by Lauren classification and other baseline characteristics. The effect of adding radiotherapy on disease-free survival (DFS) and overall survival (OS) differed according to Lauren classification, with an interaction at a significance level of *p* = 0.04 for DFS (32). The second significant article is the 2008 article “Overexpression of GRP78 and GRP94 are markers for aggressive behavior and poor prognosis in gastric carcinomas” written by Zheng H. et al. (173 citations). The study, conducted using multivariate analysis, revealed that factors such as patient age, tumor extent, presence of lymphatic invasion, lymph node metastasis, stage of the disease according to the Union Internationale Contre le Cancer classification, and tumor type according to the Lauren classification, have a significant impact on the prognosis of carcinoma (*p* < 0.5) (33). The third most cited publication in this topic is “CircCUL2 regulates gastric cancer malignant transformation and cisplatin resistance by modulating autophagy activation via miR-142-3p/ROCK2” by Peng L. et al. (169 citations). In this study circCUL2 gene expression level showed no association with Lauren classification (34) (Table 3).

The top 3 scientific journals are devoted to pathology and oncology, publishing research on the development and diagnosis of cancer. For example, Pathology - Research and Practice (8 publications and a total of 116 citations) explores diagnostic and prognostic markers, including their application in the clinic, Tumor Biology (8 articles with a total of 227 citations) investigates molecular and cellular processes in tumors, including the role of biomarkers in the progression of gastric cancer, and Human Pathology (5 articles that have garnered 241 citations) publishes papers on histopathological correlations, which is important for linking molecular changes with morphology (Figures 7A,D). Since the Lauren classification determines the treatment strategy (e.g., intestinal type is more responsive to chemotherapy, while diffuse type requires targeted approaches), studies in these journals help predict response to therapy (e.g., HER2-positive tumors are sensitive to trastuzumab), determine tumor aggressiveness (e.g., loss of E-cadherin in diffuse type is associated with a worse prognosis), and develop personalized treatment based on molecular profiling. These journals provide key data on the relationship between biomarkers, histological subtype (Laurеn) and clinical outcomes, making them indispensable for researchers and clinicians working in the field of gastric cancer.

The author’s top three most popular keywords were “gastric cancer” (94 mentions), “prognosis” (68 mentions), “immunohistochemistry” (20 mentions) (Figure 9A). Based on the frequency of occurrence of terms and their temporal distribution, several key trends can be identified. The term “biomarker” is the most relevant at present. The search for new biomarkers that will help in the early detection of malignant tumors, assessment of relapses, prognosis and individualized approach to treatment will continue for many decades (8, 14). Modern research confirms that molecular biomarkers play a key role in prognostication and personalization of treatment of gastric cancer (35–37). The term “microsatellite instability” peaked in 2020, its popularity likely due to reliability of this biomarker in gastric cancer (38, 39). “Lauren classification” is the third trending topic (Figure 10B). In recent decades, criteria-based morphological classification of gastric cancer has been Lauren often used to understand the diagnosis, treatment and prognosis of gastric cancer (40). As already mentioned, according to the classification Lauren, gastric adenocarcinomas are divided into intestinal, diffuse, mixed types. They differ not only in morphology, but also in epidemiology, progression pattern, genetics and clinical picture (41, 42). To date, it has been established prognostic value of multiple gastric cancer biomarkers in the context of the Lauren classification (43–47).

Current research in gastric cancer is focused on advanced patient stratification through a combination of traditional (HER2, PD-L1) and novel biomarkers (CLDN18.2, EBV, TMB). Contemporary chemoimmunotherapy approaches are biomarker-driven, with HER2, PD-L1, and Claudin18.2 being the most clinically significant targets (48, 49). MSI status, EBV infection, and TMB levels have demonstrated promising prognostic value (50–53). The study by Wei, J. et al. confirmed a significant association of HER2 positivity with the intestinal subtype according to Lauren (*p* = 0.005), which is consistent with the literature (10–30% vs. < 5% for the diffuse type) (54). Despite the usually better prognosis of the intestinal type, HER2/cortactin co-expression was an independent predictor of worse survival (HR = 1.427, *p* = 0.046), indicating an aggressive phenotype with a combination of these markers. The authors suggest considering dual targeted therapy for HER2+/cortactin+ tumors, which is especially relevant for the intestinal subtype (55). As shown in a study on patients from Morocco, MSI-H status shows a strong association with the Lauren intestinal subtype (*p* < 0.001) and an improved prognosis, which confirms the need to integrate classical histological classification with modern molecular markers for patient stratification. Results support the use of immunotherapy for MSI-H GC (51). In a retrospective study of 1,144 patients by Chen et al., miR-141-3p was identified as an independent prognostic factor and promising biomarker for Lauren classification (56). Tumor-infiltrating lymphocyte (TIL) density assessed on H&E slides has proven to be a reliable biomarker for predicting clinical outcomes in gastric cancer patients (57). A statistical analysis of 426 patients who underwent radical gastrectomy revealed that CLDN18.2 expression correlated with TNM stage, Lauren classification, and HER2 status (36). Recent evidence establishes EBV as a robust biomarker for gastric cancer immunotherapy (58, 59). PD-L1 expression levels remain a widely used clinical biomarker for predicting immunotherapy response (59) and serve as a key prognostic indicator in gastric cancer (60). Circulating tumor cells (CTCs) have been identified as a negative prognostic factor (associated with worse survival) with significant diagnostic potential (61).

Key future directions include: development of combination regimens (targeted therapy + immunotherapy) (62, 63). Implementation of machine/deep learning models and AI tools in pathology. Current AI applications include: endoscopic image recognition systems for gastric cancer diagnosis (64); radiomic models based on CECT for predicting lymphovascular invasion (LVI) in Lauren classification (65), Dual-energy CT (DECT)-based models using venous-phase iodine maps (IM) and 120-kVp equivalent mixed images (MIX) for noninvasive Lauren classification prediction (66); deep learning classifiers for Lauren subtyping on conventional H&E slides (67). Liquid biopsy has emerged as an advanced approach for comprehensive tumor burden quantification and longitudinal monitoring of molecular changes (68–71). AI technologies and liquid biopsy are transforming diagnostic and monitoring paradigms by enabling dynamic assessment of tumor heterogeneity. These advancements are driving gastric oncology into the era of precision medicine, where therapeutic decisions are based on molecular tumor profiling and real-time response monitoring. The use of nanoparticles (NP) in the diagnosis and treatment of gastric cancer (GC) is one of the most promising areas in modern oncology (72–74).

Despite significant progress in molecular classification and identification of biomarkers, their implementation in clinical practice remains limited. Further studies are needed to validate these biomarkers in large patient cohorts and develop standardized methods for their assessment. In addition, the integration of molecular data with traditional clinicopathological characteristics may lead to the creation of more accurate prognostic models and facilitate personalized treatment of patients with GC (75). It is extremely important for researchers to study current trends and key issues in this area, as well as to identify prospects for further research.

### 4.1 Strengths and limitations

Among the significant advantages, as we know, we can note that there have been no published bibliometric analyses examining studies on the prognostic value of gastric cancer biomarkers in relation to the Lauren classification. This is the first bibliometric analysis to cover the global research landscape. On this current topic over a 29-year period and reflects its current status. Among the limitations, we can highlight that our publications were obtained from a single Scopus database and only English-language research articles were analyzed, which could lead to incomplete coverage of the volume of existing literature. Database Scopus is one of the largest and most global databases. The volume of data from Scopus sufficient to reflect the current picture of world research in this area.

## 5 Conclusion

Studies of the prognostic value of gastric cancer biomarkers depending on Lauren’s classification are actively developing. The Lauren classification continues to be an important tool for patient stratification, and the study of new prognostic biomarkers contributes to improved diagnostics and personalized approaches to therapy. China is the leader in the study of this field, the most active research center is Sungkyunkwan University School of Medicine, the most influential researcher is Zhang J., the most publishing journal Pathology - Research and practice. The main trends include the study of microsatellite instability, biological markers and their prognostic value. This analysis confirms the relevance of the topic and the growing interest of the scientific community in the study of the prognostic significance of biomarkers depending on the Lauren classification, provides useful guidelines researchers and clinicians in the current research landscape and promising directions for future scientific research.

## Data Availability

The raw data supporting the conclusions of this article will be made available by the authors, without undue reservation.
